# Seroprevalence and associated risk factors of human brucellosis among occupational population in Binzhou, China: a cross-sectional study

**DOI:** 10.3389/fpubh.2026.1834169

**Published:** 2026-04-29

**Authors:** Jie Wu, Zhidong Wei, Lu Zhang, Zhe Liu

**Affiliations:** 1 Department of Infectious Disease Control, Binzhou Center for Disease Control and Prevention, Binzhou, Shandong, China; 2 Zouping Center for Disease Control and Prevention, Binzhou, Shandong, China

**Keywords:** Brucella species, epidemiological characteristics, occupational population, risk factors, seroprevalence

## Abstract

**Background:**

Brucellosis remains a global public health challenge, particularly in regions with high levels of livestock farming like Binzhou. This study aims to investigate the seroprevalence and associated risk factors of brucellosis among occupational populations in Binzhou.

**Methods:**

From January to December 2024, a cross-sectional study among occupational populations was conducted in Binzhou, China. A total of 1,312 participants were recruited through a multistage cluster sampling strategy. All participants underwent serological testing for brucellosis and completed a structured questionnaire. The Rose Bengal Plate Test (RBPT) was used as a screening test, followed by confirmatory Standard Tube Agglutination Test (SAT), with a titer ≥1:100 considered positive. Univariable and multivariable logistic regression were used to identify risk factors associated with seropositivity.

**Results:**

The mean age of the 1,312 participants was 56.85 ± 12.78 years. The sample comprised 952 males and 360 females. The overall seroprevalence of brucellosis was 4.73% (62/1,312), Wudi County exhibited the highest seroprevalence at 11.39% (27/237), which was significantly higher than that of other regions (*χ*^2^ = 36.967, *p* < 0.001). The seropositivity rate among males (5.5%) was also significantly higher than that among females (2.8%) (*χ*^2^ = 4.181, *p* = 0.041). Five *Brucella* strains were isolated and identified as *B. melitensis*, with an isolation rate of 8.06% (5/62). Having a history of contact with brucellosis patients (OR = 2.412, 95% CI: 1.181–4.924), having external injuries when in contact with livestock (OR = 2.298, 95% CI: 1.093–4.833), and sharing water sources (OR = 1.848, 95% CI: 1.002–3.410) were three independent risk factors associated with brucellosis infection within the occupational population.

**Conclusion:**

The seroprevalence of brucellosis among the occupational population in Binzhou is high and is primarily driven by *B.melitensis*. The risk is strongly associated with work-related injuries, contact with infected individuals and sharing water sources. Targeted interventions, including occupational safety training, protective equipment, health education, and enhanced targeted surveillance in high-risk areas, are urgently needed.

## 1 Introduction

Brucellosis is a highly prevalent zoonotic infectious disease caused by bacteria of genus *Brucella*, *B.melitensis* and *B.abortus* are the most common species that cause human infection followed by *B*.suis and *B. canis* (1). Transmission to humans occurs principally through direct contact with infected animals or the ingestion of contaminated animal products (2, 3). It remains a global public health challenge, particularly in regions with high levels of livestock farming, where people in direct contact with animals are at an elevated risk of infection (4). Brucellosis is typically associated with atypical symptoms such as fever, fatigue, musculoskeletal pain, and night sweats, often leading to misdiagnosis and chronic disease if left untreated (5). The disease can lead to significant morbidity and disability, affecting the physical, psychological, and economic well-being of individuals and posing a substantial burden on healthcare systems (6).

Human brucellosis, although preventable, has been classified as one of the top neglected zoonoses worldwide by the World Health Organization (7), and remains endemic in many parts of the world. From 2006 to 2019, the number of reports of brucellosis worldwide gradually increased from 53 countries to 97, and the reported incidence rate could reach as high as 293.103 per 100,000 people (8). It was estimated that the number of new cases increased from 500,000 to 2.1 million annually (9, 10), which could cause considerable economic losses, physical as well as mental impact, particularly among occupational groups. The mean total cost per patient was 1,060 USD in Western Iran (11) and the annual median losses due to human brucellosis were estimated to be 10.46 million USD in India, and the disability-adjusted life years(DALYs) in occupational groups(0.29 [95% UI 0.08–0.70]) were much higher than those in the non-occupational population(0.13 [95% UI 0.06–0.18]) (12). Despite this substantial global burden, investigations of brucellosis infection have mainly focused on serological testing through sampling in specific areas, and seropositivity rates vary greatly among different countries and regions. For instance, a comprehensive meta-analysis conducted in Turkey reported a pooled seroprevalence of 4.5% (95% CI: 3.8%–5.3%) among 51,560 individuals, with significantly higher rates observed in rural areas (8.0%) and high-risk occupational groups (9.9%) (13). A cross-sectional survey among abattoir workers in South Africa revealed that low-throughput abattoirs were identified as a risk factor, with a seroprevalence of 29.3% among workers compared with 12.7% in high-throughput abattoirs (14). Similarly, in Tanzania, a recent study conducted in a multi-herd ranch system reported a human seroprevalence of 41%, with close contact with animals (OR = 7.20, 95% CI: 1.97–36.31) and handling of parturition or aborted fetuses (OR = 2.37, 95% CI: 1.01–5.58) identified as significant risk factors (3). In West Africa, a serosurvey found that 9.5% of abattoir and dairy farm workers were seropositive for Brucella, and the consumption of unboiled milk was associated with significantly increased odds of seropositivity (OR = 3.79, 95% CI: 2.34–6.13) (15). These striking variations in seroprevalence across different geographical and occupational contexts underscore the complex, multifactorial nature of brucellosis transmission. Moreover, there is a notable lack of in-depth surveys targeting the key occupational groups exposed to brucellosis, particularly in regions where livestock farming is a primary economic activity.

This research gap is particularly evident in China, where the majority of high-risk occupational populations reside in rural areas with limited educational attainment. A national serological survey conducted in China’s endemic areas reported that only 8.93% of high-risk occupational participants had completed senior high school or above (16). Their awareness of brucellosis transmission routes and preventive measures is often inadequate. Some studies have shown that the awareness rate of brucellosis-related knowledge among occupational workers is low, and the adoption of protective practices remains suboptimal: the standardized utilization rate of personal protective equipment among China’s high-risk occupational populations was only 20.13%, with even lower rates observed among those with lower educational levels (17). This lack of knowledge and poor health literacy further elevates their risk of infection and hinders timely healthcare-seeking behavior. Therefore, understanding the seroepidemiological status and the specific knowledge gaps among this vulnerable population is critical for designing effective, targeted health education interventions.

Binzhou, located in Shandong Province, is an important agricultural hub where livestock farming, especially cattle, sheep, and goats, is a significant part of the local economy. From 2005 to 2015, the annual incidence rate of human brucellosis in Binzhou was 2.40 per 100,000 people. It reached its peak in 2015 (7.39 per 100,000 people), and then showed a decreasing trend year by year, but still remained at a relatively high level (18, 19). A large proportion of the rural population is engaged in occupationally high-risk activities such as animal husbandry, slaughtering, milking, livestock trading, and veterinary services. Despite the high potential for brucellosis transmission in this setting, no previous seroepidemiological study has been conducted among occupational populations in Binzhou to assess the seroprevalence of brucellosis or to identify associated risk factors. Therefore, this study was designed to fill this research gap by providing baseline data on the serological status and risk factors for brucellosis among high-risk occupational groups in Binzhou.

This is the first city-wide serological surveillance and systematic risk factor study of brucellosis among occupational populations in Binzhou. The serological surveillance can identify infected individuals who are asymptomatic or have not sought medical care and enable early diagnosis and timely treatment, thereby preventing disease chronicity. Furthermore, combining serological data with questionnaire surveys on occupational tasks, hygiene practices, and protective behaviors helps identify local transmission drivers, which are expected to provide essential data support and a practical reference for local health authorities in developing evidence-based prevention and control strategies.

## 2 Materials and methods

### 2.1 Study design

This was a cross-sectional study based on a sample survey conducted by the Binzhou Center for Disease Control and Prevention from January 1, 2024 to December 31, 2024, to identify the seroprevalence and associated risk factors among the occupational population.

### 2.2 Study population and the inclusion and exclusion criteria

Data on the occupational population were obtained from the Binzhou Animal Husbandry and Veterinary Management Service Center. We collected study population according to the following inclusion criteria:(1) the individuals’ current address was in Binzhou; (2) people engaged in related occupations for at least 1 year by December 31, 2023; (3) occupational groups refer to those engaged in occupations such as grazing, breeding, transportation, slaughtering, meat product sales (for cattle and sheep), meat product processing (including personnel processing beef and mutton in restaurants), feeding other characteristic animals (such as minks and foxes), fur packaging processing, dairy product processing, veterinarians. Exclusion criteria were as follows: (1) people who have been engaged in jobs for less than 1 year; (2) individuals who could not be contacted; (3) patients with brucellosis who were reported before 2024 or patients whose clinical diagnosis progressed to the chronic stage.

### 2.3 Sample size and sampling procedure

The sample size of 1,298 was determined using the formula as follows, $n=\frac{z^{2}\cdot p\cdot (1-p)}{d^{2}}$ , *n* = required sample size, z = z-score (based on the desired confidence level, in this study 1.96 for 95% confidence), p = estimated proportion of the attribute present in the population (a brucellosis seroprevalence of 3.5% in this study), 1-p = estimated proportion of the attribute not present in the population, d = margin of error (as a decimal of 0.01).

A multistage cluster sampling strategy was employed to select participants. In the first stage, all 7 districts of the study region were included. In the second stage, within each district, three townships were selected using probability proportional to size (PPS) sampling, where the size measure was the number of occupational population in each township. In the third stage, within each sampled township, two administrative villages or large-scale breeding farms were randomly selected as the final sampling clusters. All eligible individuals from the occupational population within each selected village or farm were enrolled in the study. A total of 1,312 participants were ultimately enrolled.

### 2.4 Laboratory tests

Venous blood (3–5 mL) was collected from each participant. The Rose Bengal Plate Test (RBPT) was used for initial screening, and samples showing any visible agglutination were considered positive for screening. According to the manufacturer’s specifications (Qingdao Zhongchuang Huike Biotechnology Co., Ltd., China), the RBPT used in this study has a sensitivity of 100% and a specificity of >97%. For those with positive results, the samples were further subjected to the serum agglutination test (SAT). A SAT titer ≥1:100 (++) was defined as a laboratory-confirmed case of human brucellosis. The SAT kit (same manufacturer) has a specificity of 100% at this diagnostic cutoff. All serological tests were performed in the laboratory of Binzhou Center for Disease Control and Prevention. The culture and identification of *Brucella* bacteria, as well as the diagnosis of human brucellosis were based on the “Diagnosis for brucellosis (WS 269–2019)” issued by National Health Commission of the People’s Republic of China in 2019 (20).

### 2.5 Data collection and quality control

The questionnaire was adapted from the brucellosis case investigation form of the *National Brucellosis Surveillance Work Plan* (National Health and Family Planning Commission of China, (21)). Based on this foundation, additional items on brucellosis-related knowledge and potential risk factors were added by the research team. A pilot test of the revised questionnaire was conducted in a breeding farm in one county to assess clarity, feasibility, and completeness; minor wording adjustments were made based on the pilot results.

A questionnaire survey was then conducted among all selected occupational populations using this unified epidemiological case investigation form. The final questionnaire covered general information (gender, age, and occupation), epidemiological history, and brucellosis-related knowledge.

Given that Binzhou City has seven administrative districts, seven investigation teams were formed, each consisting of two experienced field epidemiologists and two laboratory professionals. Each team was assigned to one district and conducted on-site questionnaire surveys and sample collection at the participants’ workplaces or residences.

The questionnaire survey was administered by designated staff from the Binzhou Center for Disease Control and Prevention. All personnel involved in the field investigation received standardized training, and only those who passed the assessment were permitted to conduct the fieldwork. Each completed questionnaire was reviewed by the investigators on the same day to identify and correct any missing items or errors. In addition, quality assurance measures were implemented at every stage of the laboratory processes, with regular checks to ensure strict adherence to standard operating procedures.

### 2.6 Statistical analysis

Statistical analyses were performed using R software (version 4.3.1, R Core Team, Vienna, Austria). Continuous variables are presented as mean ± standard deviation, and categorical variables as frequencies and percentages (%). The chi-square test was used for between-group comparisons of categorical variables, and the independent-samples *t*-test for continuous variables. Univariate and multivariate logistic regression models were employed to identify risk factors associated with brucellosis seropositivity, with results expressed as odds ratios (ORs) and 95% confidence intervals (CIs). A *p value* of less than 0.05 was considered statistically significant.

## 3 Results

### 3.1 Demographic characteristics of occupational population

There were a total of 13,657 occupational individuals engaged in occupations related to brucellosis by December 31, 2023 in Binzhou. Huimin County (3,951, 28.93%), Zouping City (3,210, 23.50%), Wudi County (2,125, 15.56%), Yangxin County (1,929, 14.12%), and Bincheng District (1,553, 11.37%) have a large number of occupational groups, accounting for 93.49% of the city’s total (Table 1). In Binzhou, there are 12,765 male and 892 female occupational groups for brucellosis, with a male-to-female ratio of approximately 14.3:1; there is a statistically significant difference in the gender distribution of occupational groups for brucellosis in different regions (*χ*^2^ = 1,211.112, *p* < 0.001). Table 2 shows the occupational distribution of participants across the seven counties/districts. The most common occupation was livestock raising and grazing (12,345, 90.39%), followed by breeder (1,044, 7.64%).

**Table 1:** Distribution of the occupational population in Binzhou (*N* = 13,657).

Region	Male (*n*, %)	Female (*n*, %)	Total (*n*, %)
Bincheng District	1,484 (95.56)	69 (4.44)	1,553
Huimin County	3,866 (97.85)	85 (2.15)	3,951
Yangxin County	1,880 (97.46)	49 (2.54)	1,929
Wudi County	2,099 (98.78)	26 (1.22)	2,125
Zhanhua District	453 (98.05)	9 (1.95)	462
Boxing County	405 (94.85)	22 (5.15)	427
Zouping City	2,578 (80.31)	632 (19.69)	3,210
Total	12,765 (93.47)	892 (6.53)	13,657

**Table 2:** Occupational distribution of participants in Binzhou (*N* = 13,657).

Occupation	Bincheng District	Huimin County	Yangxin County	Wudi County	Zhanhua District	Boxing County	Zouping City	Total
Breeder	133	184		509	116		102	1,044
Livestock raising and grazing	1,352	3,703	1,881	1,616	296	427	3,070	12,345
Livestock trading		2	28				4	34
Meat or dairy product processing/sales	1	12			14		10	37
Slaughter	49	11	16	0	27	0	7	110
Veterinarian	18	39	4		9		17	87
Total	1,553	3,951	1,929	2,125	462	427	3,210	13,657

### 3.2 Epidemiological characteristics of participants

Among the 13,657 individuals in the occupational population, 1,312 participants were enrolled through multistage cluster sampling to undergo brucellosis testing and complete a questionnaire survey. The mean age of the 1,312 participants was 56.85 ± 12.78 years, with 952 males and 360 females. As shown in Tables 3, 4, the largest number of participants came from Wudi County (237, 18.07%), followed by Huimin County (229, 17.45%) and Zouping City (211, 16.08%). Regarding educational attainment, 52.2% (685/1,312) of participants had primary school education or below, 45.2% (593/1,312) had junior or senior high school education, and only 2.6% (34/1,312) held a university diploma or above.

**Table 3:** Seroprevalence of brucellosis among occupational participants in Binzhou (*N* = 1,312).

County/District	Number of seropositive individuals (*n*)	Seroprevalence (%)
Bincheng District (*n* = 210)	10	4.76
Boxing County (*n* = 113)	5	4.42
Huimin County (*n* = 229)	12	5.24
Wudi County (*n* = 237)	27	11.39
Yangxin County (*n* = 208)	2	0.96
Zhanhua District (*n* = 104)	4	3.85
Zouping City (*n* = 211)	2	0.95
Total (*N* = 1,312)	62	4.73

**Table 4:** Distribution of brucellosis infection among occupational population in Binzhou (*N* = 1,312).

Variable	Seropositive, (*n*, %)	Seronegative, (*n*, %)	*χ*^2^/t	*p* value
Gender				
Male (*n* = 952)	52 (5.46)	900 (94.54)	4.181	0.041
Female (*n* = 360)	10 (2.78)	350 (97.22)		
Education				
Primary school and below (*n* = 685)	34 (4.96)	651 (95.04)	0.340	0.843
Junior and senior high school (*n* = 593)	26 (4.38)	567 (95.62)		
University diploma or above (*n* = 34)	2 (5.88)	32 (94.12)		
Age (years), mean ± SD	54.48 ± 12.62	56.95 ± 12.79	1.483	0.138
Family population, mean ± SD	3.45 ± 1.74	3.46 ± 1.74	0.036	0.971
Total (*N* = 1,312)	62 (4.73)	1,250 (95.27)		

### 3.3 Seroprevalence of occupational population

Of the 1,312 occupational populations, 62 individuals were seropositive, with a seroprevalence of 4.73% (62/1,312). There was a statistically significant difference in the seroprevalence of occupational groups for brucellosis among the 7 regions in Binzhou City (χ^2^ = 36.967, *p* < 0.001). Wudi County had the highest seroprevalence of 11.39% (27/237), much higher than that in other regions (Table 3).

As shown in Table 4, there was a statistically significant difference in the seroprevalence among different gender groups of occupational populations (*χ*^2^ = 4.181, *p* = 0.041). The seroprevalence of brucellosis was significantly higher in males (5.46%, 52/952) than in females (2.78%, 10/360). Regarding educational level, seroprevalence was 4.96% (34/685) among participants with primary school education or below, 4.38% (26/593) among those with junior or senior high school education, and 5.88% (2/34) among those with a university diploma or above; these differences were not statistically significant (*χ*^2^ = 0.340, *p* = 0.843). No significant differences were observed in age or family population size between seropositive and seronegative individuals (*p* > 0.05).

Table 5 presents the association between brucellosis seroprevalence and occupational categories. Slaughter had the highest seroprevalence (13.64%, 9/66), which was significantly higher than other occupational groups (*χ*^2^ = 12.256, *p* < 0.001). Veterinarians showed a seroprevalence of 9.09% (3/33), followed by breeders (5.81%, 5/86) and those engaged in livestock-raising and grazing (4.86%, 52/1,071). No statistically significant differences were found among these occupations.

**Table 5:** Seropositivity rate of brucellosis among different occupational groups (*N* = 1,312).

Occupation	Number of seropositive individuals (*n*, %)	Number of seronegative individuals (*n*, %)	*χ* ^2^	*p* value
Livestock raising and grazing (*n* = 1,071)	52 (4.86)	1,019 (95.14)	0.218	0.641
Slaughter (*n* = 66)	9 (13.64)	57 (86.36)	12.256	<0.001
Breeder (*n* = 86)	5 (5.81)	81 (94.19)	0.242	0.623
Veterinarian (*n* = 33)	3 (9.09)	30 (90.91)	1.433	0.231
Other (*n* = 56)	2 (3.22)	54 (93.48)	0.173	0.677
Total (*N* = 1,312)	62 (4.73)	1,250 (95.27)		

### 3.4 Species of *Brucella* identified

Blood samples from 62 individuals who tested positive for brucellosis were collected for *Brucella* strain isolation and identification. *Brucella* strains were successfully isolated from 5 of these seropositive individuals, yielding a strain culture rate of 8.06%. All five strains were *B. melitensis*, with three identified as Biovar 3 and two as Biovar 1.

### 3.5 Risk factors of occupational population

Univariate logistic regression was conducted to identify risk factors associated with brucellosis seropositivity (Table 6). Four factors were significantly associated with an increased risk of infection: having external injuries when in contact with livestock (OR = 3.374, 95% CI: 1.730–6.583), contact with brucellosis patients (OR = 3.379, 95% CI: 1.768–6.457), hunting and consuming wild animals (OR = 5.175, 95% CI: 1.076–24.899), and sharing water sources (OR = 2.000, 95% CI: 1.094–3.656). No significant associations were observed for contact with excretions of diseased animals, separate use of cooked and raw kitchenware, livestock farming at home, or keeping pets at home (*p* > 0.05). Contact with brucellosis patients in this study mainly referred to household/family contact.

**Table 6:** Univariate logistic regression analysis of risk factors in occupational population (*N* = 1,312).

Variables	*B*	SE	Wald	*p* value	OR (95% CI)
Contact with the excretions (such as urine and feces) of diseased animals (yes = 1, no = 0)	−0.301	0.262	1.318	0.251	0.740 (0.443, 1.237)
Having external injuries when in contact with livestock (yes = 1, no = 0)	1.216	0.341	12.726	<0.001	3.374 (1.730, 6.583)
Hunting and consuming wild animals (yes = 1, no = 0)	1.644	0.802	4.206	0.040	5.175 (1.076, 24.899)
Contact with patients with brucellosis (yes = 1, no = 0)	1.218	0.330	13.578	<0.001	3.379 (1.768, 6.457)
Shared water sources (yes = 1, no = 0)	0.693	0.308	5.076	0.024	2.000 (1.094, 3.656)
Separate cooked and raw kitchenware (yes = 1, no = 0)	−0.071	0.287	0.061	0.806	0.932 (0.531, 1.635)
Livestock farming at home (yes = 1, no = 0)	0.219	0.410	0.287	0.592	1.245 (0.558, 2.779)
Keep and pets at home (yes = 1, no = 0)	0.031	0.261	0.014	0.906	1.031 (0.618, 1.722)

These four significant variables were then entered into a multivariate logistic regression model to identify independent risk factors (Table 7). After adjusting for potential confounders, three factors remained significantly associated with brucellosis seropositivity: contact with brucellosis patients (OR = 2.412, 95% CI: 1.181–4.924, *p* = 0.016), having external injuries when in contact with livestock (OR = 2.298, 95% CI: 1.093–4.833, *p* = 0.028), and sharing water sources (OR = 1.848, 95% CI: 1.002–3.410, *p* = 0.049).

**Table 7:** Multivariate logistic regression of risk factors in occupational population (*N* = 1,312).

Variables	*B*	SE	Wald	*p* value	OR (95% CI)
Having external injuries when in contact with livestock (yes = 1, no = 0)	0.832	0.379	4.814	0.028	2.298 (1.093, 4.833)
Hunting and consuming wild animals (yes = 1, no = 0)	1.046	0.845	1.532	0.216	2.846 (0.543, 14.906)
Contact with patients with brucellosis (yes = 1, no = 0)	0.880	0.364	5.841	0.016	2.412 (1.181, 4.924)
Shared water sources (yes = 1, no = 0)	0.614	0.313	3.862	0.049	1.848 (1.002, 3.410)

## 4 Discussion

This cross-sectional study provides a detailed epidemiological snapshot of brucellosis among high-risk occupational groups in Binzhou, China. The overall seroprevalence of 4.73% among the sampled occupational groups is substantial and indicates active transmission. This rate is significantly higher than the national average reported for occupational groups in some Chinese studies, which often range from 1% to 3% (22, 23), underscoring the significant and disproportionate burden of this zoonosis on individuals with direct livestock exposure. The elevated prevalence aligns with findings from other agricultural and pastoral regions globally, highlighting occupational contact as a primary driver of transmission (24).

A study on the epidemiological characteristics of brucellosis in Binzhou from 2015 to 2020 identified Zhanhua District, Yangxin County, and Wudi County as the areas with the highest incidence rates, with farmers accounting for 90.58% of reported cases (19). In our study, Wudi County had the highest seroprevalence (11.39%), which aligns with these previous observations and confirms that Wudi remains a high-risk area for brucellosis transmission. The regional differences in seroprevalence likely reflect variations in livestock density, farming practices, and levels of disease surveillance and control measures across the seven regions.

Our results demonstrated a significantly higher seroprevalence in males (5.46%) compared to females. This gender difference is consistent with the occupational nature of brucellosis. First, the number of occupational individuals in Binzhou is much higher in males (12,765) than in females (892), with a male-to-female ratio of approximately 14.3:1, which is a common phenomenon worldwide (14, 25). Second, men are primarily responsible for livestock raising and slaughtering activities in many agricultural regions. A meta-analysis of risk factors for human brucellosis found that occupation-related exposure is a significant risk factor for infection (OR = 3.31, 95% CI: 1.6–6.55) (26), which may explain the higher seroprevalence observed in males. Regarding age, the mean age of participants was 56.85 ± 12.78 years, and no significant difference was observed between seropositive and seronegative individuals. However, previous studies in Binzhou have shown that the highest number of brucellosis cases occurs in the 60–70 year age group (19). This pattern may reflect the aging agricultural workforce in rural China, where younger individuals have increasingly migrated to urban areas for employment. Educational level showed no significant association with seropositivity, although 52.2% of participants had only primary school education or below. This finding suggests that while low educational attainment may contribute to lack of awareness about brucellosis prevention, other factors such as specific occupational behaviors are more direct determinants of infection risk (26, 27). This observation is consistent with other studies reporting that behavioral factors may override the protective effect of education in high-risk occupational settings.

Among different occupational categories, slaughter had the highest seroprevalence (13.64%, 9/66), which was significantly higher than other occupational groups (*p* < 0.001). This finding is consistent with outbreak investigations from other regions of China. In a 2023 outbreak investigation at a slaughterhouse in Zigong, Sichuan Province, the risk of *Brucella* infection among slaughterhouse workers was five times higher than that of other occupational groups (RR = 5.14, 95% CI: 1.90–13.98), and “engage in the profession of slaughtering” was identified as an independent risk factor (OR = 5.87, 95% CI: 1.54–22.45) (28). The high seroprevalence among slaughterhouse workers in Binzhou likely reflects their direct and intensive contact with blood, viscera, and other tissues from infected animals during the slaughtering process. Veterinarians also showed elevated seroprevalence (9.09%), followed by breeders (5.81%). These findings are consistent with the understanding that occupational exposure to infected animals, their birth products, and contaminated environments are major transmission routes for brucellosis (26).

Of the 62 seropositive individuals, *Brucella* strains were successfully isolated from 5. All five isolates were identified as *B. melitensis*, with three classified as biovar 3 and two as biovar 1. This species and biovar distribution is consistent with the epidemiological profile of brucellosis in China (29, 30), which revealed that *B. melitensis* (particularly biovar 3) is the primary cause of human brucellosis in mainland China. While the isolation rate (8.06%) appears low, it is not uncommon in clinical *Brucella* isolation practice. A review from Iran noted that positivity rates with conventional blood culture are generally as low as 2% (31). Chinese national surveillance data from 2009 showed that 68 strains of *B. melitensis* biotype 3 were isolated nationwide, accounting for 94.7% of all isolates, but this number was extremely low compared to the 35,816 reported brucellosis cases that same year (32). This indicates that low *Brucella* isolation rates are not unique to Binzhou but represent a common challenge in brucellosis laboratory diagnosis across China and globally. The five isolates obtained in this study provide, for the first time, insights into the molecular characteristics of locally circulating strains in this region. These isolates are valuable for molecular epidemiological tracing, antimicrobial resistance surveillance, and future vaccine development. We recommend the adoption of automated blood culture systems, optimization of pre-culture processing techniques, and establishment of *Brucella* isolation capacity in provincial or regional reference laboratories to improve isolation rates in Binzhou, and even in Shandong Province.

Multivariate logistic regression analysis identified three independent risk factors for *Brucella* seropositivity. Firstly, occupational individuals who had a history of contact with brucellosis patients (OR = 2.412, 95% CI: 1.181–4.924, *p* = 0.016) may have a higher risk of getting infected with brucellosis. It is worth mentioning that contact with brucellosis patients mainly referred to household or family contact in this study. Though rare cases of human-to-human transmission have been reported including through blood transfusion, bone marrow transplantation, and sexual contact, the possibility of human-to-human transmission through close contact remains a topic of debate (33–36). Household transmission may also occur through shared living spaces and contact with contaminated materials (37). Secondly, having external injuries when in contact with livestock (OR = 2.298, 95% CI: 1.093–4.833, *p* = 0.028) highlights the importance of skin barrier integrity in preventing Brucella infection. The bacteria can enter the body through breaks in the skin, making individuals with cuts, abrasions, or other wounds particularly vulnerable when handling infected animals or their tissues. This finding underscores the importance of using personal protective equipment (gloves, protective clothing) and promptly treating any wounds before animal contact (14, 15). Finally, sharing water sources (OR = 1.848, 95% CI: 1.002–3.410, *p* = 0.049) is also a risk factor, which reflects the practice of sharing drinking water with livestock or using contaminated water sources (38). In rural farming communities, it is not uncommon for humans and animals to share water sources, which can become contaminated with *Brucella* through infected animal urine, feces, or birthing materials.

Based on the findings of this study, we recommend the following targeted interventions: firstly, providing occupational safety training and protective equipment (gloves, masks) for livestock farmers with emphasis on wound care before animal contact (26); secondly, implementing community-based health education on brucellosis transmission routes, early symptoms, and the risks of sharing water sources with livestock; thirdly, strengthening laboratory capacity in regional reference laboratories through automated blood culture systems and optimization of pre-culture sample processing techniques to improve isolation rates (39); finally, conducting targeted surveillance and intervention in high-incidence districts such as Wudi County.

## 5 Conclusion

This cross-sectional study reveals a substantial seroprevalence of brucellosis (4.73%) among high-risk occupational groups in Binzhou, highlighting ongoing active transmission. Slaughterhouse workers exhibited the highest risk, followed by veterinarians and breeders, with *B. melitensis* biovars 1 and 3 identified as the circulating strains. Household contact with brucellosis patients, presence of external injuries during livestock contact and sharing water sources with livestock are independent risk factors for *Brucella* infection. Notably, Wudi County showed the highest seroprevalence (11.39%), consistent with prior surveillance data. These findings underscore the urgent need for targeted interventions, including occupational safety training and personal protective equipment, community health education on transmission routes and water source management, enhanced laboratory capacity for *Brucella* isolation using automated systems, and focused surveillance in high-risk districts like Wudi. Addressing these factors is critical to reducing the occupational burden of brucellosis in Binzhou.

## 6 Limitations

Despite the meaningful findings, several limitations of this study should be acknowledged. First, the cross-sectional design limits our ability to establish definitive causal relationships between the identified risk factors (such as external injuries and contact with patients) and brucellosis infection; it only allows for the assessment of association. Second, although the overall sample size was substantial, the number of confirmed human *Brucella* isolates was relatively small (*n* = 5). This restricts the generalizability of the conclusions regarding the molecular epidemiology and genotypic diversity of *B. melitensis* circulating in Binzhou. Third, the collection of exposure data via structured questionnaires is subject to recall bias, which may have affected the accuracy of self-reported behaviors and histories. Additionally, the study was conducted exclusively in Binzhou, which may limit the extrapolation of these findings to populations in regions with different livestock management practices or socioeconomic backgrounds. Future research should incorporate longitudinal designs, larger-scale bacterial isolation, and molecular typing techniques to further elucidate the transmission dynamics and confirm the risk factors identified in this study.

## Data Availability

The original contributions presented in the study are included in the article/Supplementary material, further inquiries can be directed to the corresponding author.
