# Supplementary figures and images for: Seroprevalence and associated risk factors of human brucellosis among occupational population in Binzhou, China: a cross-sectional study
Source: Front Public Health. 2026 Apr 29;14:1834169. doi: 10.3389/fpubh.2026.1834169 (PMC13168158; doi:10.3389/fpubh.2026.1834169)

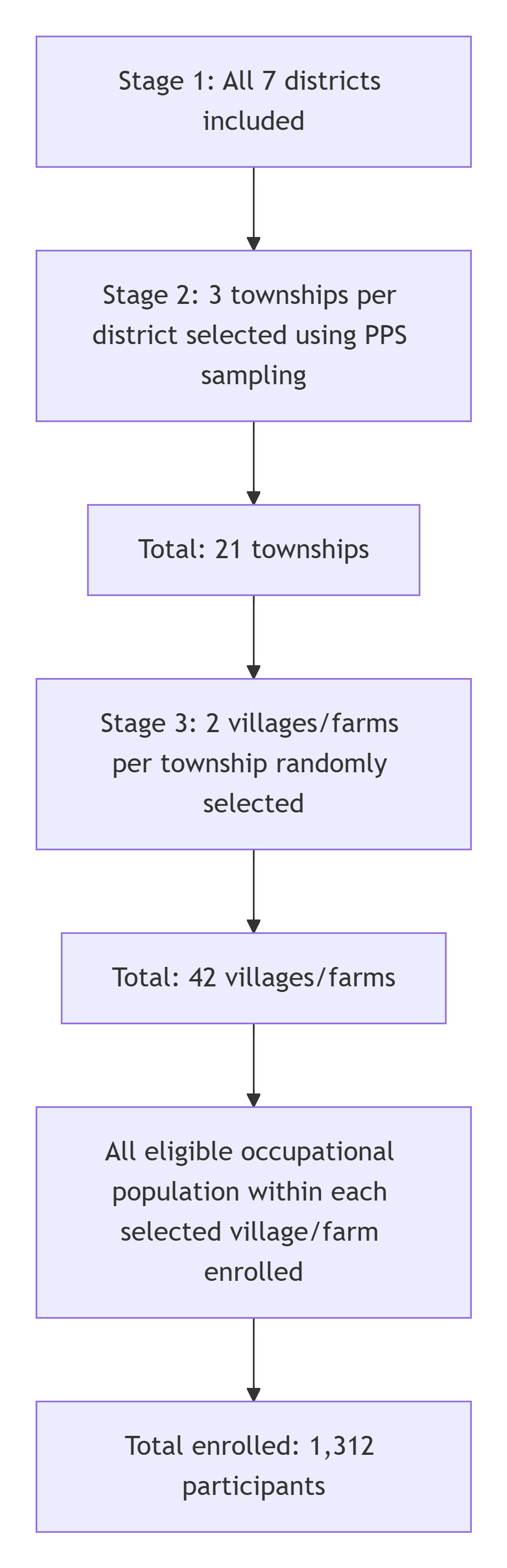

Supplement: Supplementary file 1 [file Image_1.PNG]
